# Periconception Maternal Folate Status and Human Embryonic Cerebellum Growth Trajectories: The Rotterdam Predict Study

**DOI:** 10.1371/journal.pone.0141089

**Published:** 2015-10-22

**Authors:** Irene V. Koning, Irene A. L. Groenenberg, Anniek W. Gotink, Sten P. Willemsen, Manon Gijtenbeek, Jeroen Dudink, Attie T. J. I. Go, Irwin K. M. Reiss, Eric A. P. Steegers, Régine P. M. Steegers-Theunissen

**Affiliations:** 1 Department of Obstetrics and Gynaecology, Erasmus University Medical Center, Rotterdam, the Netherlands; 2 Department of Paediatrics, subdivision of Neonatology, Sophia Children’s Hospital, Rotterdam, the Netherlands; 3 Department of Biostatistics, Erasmus University Medical Center, Rotterdam, the Netherlands; 4 Department of Radiology, Sophia Children’s Hospital, Rotterdam, the Netherlands; University of Rennes-1, FRANCE

## Abstract

We aimed to investigate whether periconceptional maternal folate status affects human embryonic cerebellar size and growth trajectories. In a prospective periconceptional cohort participants filled out questionnaires and received weekly transvaginal 3D-ultrasounds between 7+0 and 12+6 weeks gestational age (GA). Viable non-malformed singleton pregnancies were selected for cerebellar measurements; transcerebellar diameter, (TCD), left and right cerebellar diameters (LCD, RCD). Linear mixed models were performed to estimate associations between questionnaire data on the timing of maternal folic acid supplement initiation and longitudinal cerebellar measurements as a function of crown-rump length (CRL) and GA. Maternal red blood cell folate concentrations were analysed before 8 weeks GA to validate the associations. A total of 263 serial high quality three-dimensional ultrasound scans of 135 pregnancies were studied. Preconceptional compared to postconceptional initiation of folic acid use was associated with slightly larger cerebellar diameters per millimetre increase of CRL (TCD: β = 0.260mm, 95%CI = 0.023–0.491, p<0.05; LCD: β = 0.171mm, 95%CI = 0.038–0.305, p<0.05; RCD: β = 0.156mm, 95%CI = 0.032–0.280, p<0.05) and with proportional cerebellar growth (TCD/CRL:β = 0.015mm/mm, 95%CI = 0.005–0.024, p<0.01; LCD/CRL:β = 0.012mm/mm, 95%CI = 0.005–0.018, p<0.01; RCD/CRL:β = 0.011mm/mm, 95%CI = 0.005–0.017, p<0.01). Cerebellar growth was significantly highest in the third quartile of maternal red blood cell folate levels (1538–1813 nmol/L). These first findings show that periconceptional maternal folate status is associated with human embryonic cerebellar development. Implications of these small but significant variations for fetal cerebellar growth trajectories and the child’s neurodevelopmental outcome are yet unknown and warrant further investigation.

## Introduction

Last decades our understanding of the complex functions of the human cerebellum has expanded immensely. Previous literature demonstrated cerebellar involvement in motor and non-motor functions including perception, cognition and emotion [1,2]. Yet, prenatal studies of the cerebellum have mainly been focussing on abnormal cerebellar development and morphology in the second half of pregnancy when ultrasonography landmarks of development can be visualized [3–5]. However, the first trimester of pregnancy is known to be essential for organogenesis and deviations in growth and development are likely to originate during this particular period. Nowadays, ultrasound measurements of embryonic structures are more easily accessible through the improvements of ultrasound techniques. Growth charts of embryonic brain structures including the cerebellum were created with measurements performed between 7^+0^ and 12^+6^ weeks of gestation using three-dimensional ultrasound and virtual reality ultrasound visualization [6]. Three-dimensional techniques provide very precise information on variations in first trimester cerebellar growth trajectories.

Development of the cerebellum starts from the 6^th^ week of gestation and extends to the first years of life [7]. Due to this protracted process, the cerebellum is extremely vulnerable to disturbances in its development caused by a complex interplay of genetic and environmental factors [8,9]. Perinatal environmental and genetic risk factors act at particular time windows when cerebellar development is most vulnerable to derangements [10]. Maternal exposures and conditions during the periconceptional period are known to influence embryonic growth [11–13]. These factors may also affect embryonic cerebellar growth with potential implications for fetal cerebellar growth and neurodevelopmental functions in later life [14,15].

Periconceptional maternal folate status plays a vital role in biological processes involved in cellular growth and differentiation [16]. Maternal folate deficiency is associated with a broad spectrum of reproductive failures [17]. In addition, maternal periconceptional folic acid supplement use is known to reduce the risk of congenital anomalies such as neural tube defects and probably also congenital heart disease and oral facial clefts and offspring born small for gestational age (SGA) [18–20]. Furthermore adequate maternal periconceptional folic acid use seems beneficial to early and late human neurodevelopmental outcome and may reduce the risk of neurodevelopmental disorders including autism spectrum disorder in which the cerebellum seems to play a key role [21–26]. Animal models show dramatic alterations in both prenatal and postnatal cerebellar growth and development when derangements of the folate pathway and changes in folate status occurred [27,28]. So far no human studies reported on consequences of maternal folic acid supplement use on the growth and development of the human cerebellum. From this perspective we aim to investigate whether the timing of maternal folic acid supplement initiation during the periconceptional period as measure of periconceptional maternal folate status affects human embryonic cerebellar size and growth trajectories in pregnancies ending in live births without congenital malformations.

## Materials and Methods

### Study Population

This study is embedded in the Rotterdam Predict study, an ongoing prospective periconceptional cohort investigating the influence of gene-environment interactions and underlying mechanisms on reproductive, (extra-)embryonic and pregnancy outcome at the Erasmus MC University Medical Centre, Rotterdam, The Netherlands [13]. Pregnant women of at least 18 years of age with a gestational age (GA) of less than 8 weeks were eligible for participation and were followed until 1 year after delivery. All pregnant women and their partners gave written informed consent before participation. The Central Committee of Human Research in The Hague and the regional Medical Ethical and Institutional Review Board of the Erasmus MC University Medical Centre approved the study (MEC 2004–227, 15 October 2004).

We selected singleton pregnancies conceived spontaneously or through assisted reproductive techniques using biological oocytes from the participating mother with reliable GA only. GA was defined as reliable when; 1) GA was calculated using the first day of the last menstrual period (LMP) confirmed by first trimester ultrasound evaluation for spontaneously conceived pregnancies with regular menstrual cycles of approximately 28 days and was adjusted when the cycle was prolonged (>31 days) or shortened (<25 days). 2) In pregnancies assisted with in vitro fertilization (IVF), intracytoplasmic sperm injection (ICSI) and intra-uterine insemination, GA was calculated from the date of oocyte retrieval plus 14 days, from the day of embryo transfer plus 17 or 18 days cryopreserved embryo transfers, or from insemination date plus 14 days respectively.

In our statistical analyses, we used Crown-rump Length (CRL) as outcome variable. Therefore when there was no reliable recall of the (LMP) or CRL measurements differed more than 7 days from the expected GA, pregnancies were dated on CRL and consequently excluded. Because this study aimed to investigate the initiation of periconceptional maternal folic acid supplement intake in pregnancies ending in live births without congenital malformations, we also excluded pregnancies ending in miscarriages, ectopic pregnancy and termination of pregnancy or pregnancies with adverse foetal outcome including minor and major congenital malformations, foetal or neonatal death. When data from questionnaires or maternal folic acid status was missing, pregnancies were also excluded from further analysis.

### Measurements

The study visit before 8 weeks GA was used to verify self-administered questionnaires to obtain information on the current pregnancy with regard to maternal age, preconceptional weight and height, ethnicity, educational level, medical history, gynaecological and obstetric history, familial hereditary or congenital disorders and diseases, diet, lifestyle and the use of folic acid and/or other (multi)vitamin supplements. The accuracy of the self-reported questionnaires was enhanced by an intake consultation at enrolment discussing unclear topics and questions. Follow-up on pregnancy, fetal and infant outcomes comprised of questionnaires validated by the structural ultrasound scan at 20 weeks GA and delivery reports. Periconceptional was defined as the period of 4 weeks before until 8 weeks after conception. Timing of initiation of folic acid use was defined as the initiation at any time before (pre) or after (post) conception of (multi)vitamin supplements containing folic acid either the standard low dosage of 0.4–0.5 mg/day or high dosage of 5mg/day. Timing of folic acid initiation served as measure for duration of folic acid supplement use and implicitly the folate status during the first trimester of pregnancy. In a small subgroup (n = 57) a venous blood sample was collected at enrolment for determination of red blood cell folate according to the study protocol representing maternal folate status of 2–4 months previously [29]. Thereby red blood cell folate reflects the folate status of the periconceptional period and specifically the preconceptional period. As red blood cell folate is significantly higher in preconceptional folic acid users (p<0.01), we demonstrated that red blood cell folate concentration is an accurate indicator of the timing of initiation and duration of folic acid supplement use in our study population.

**Fig 1:**
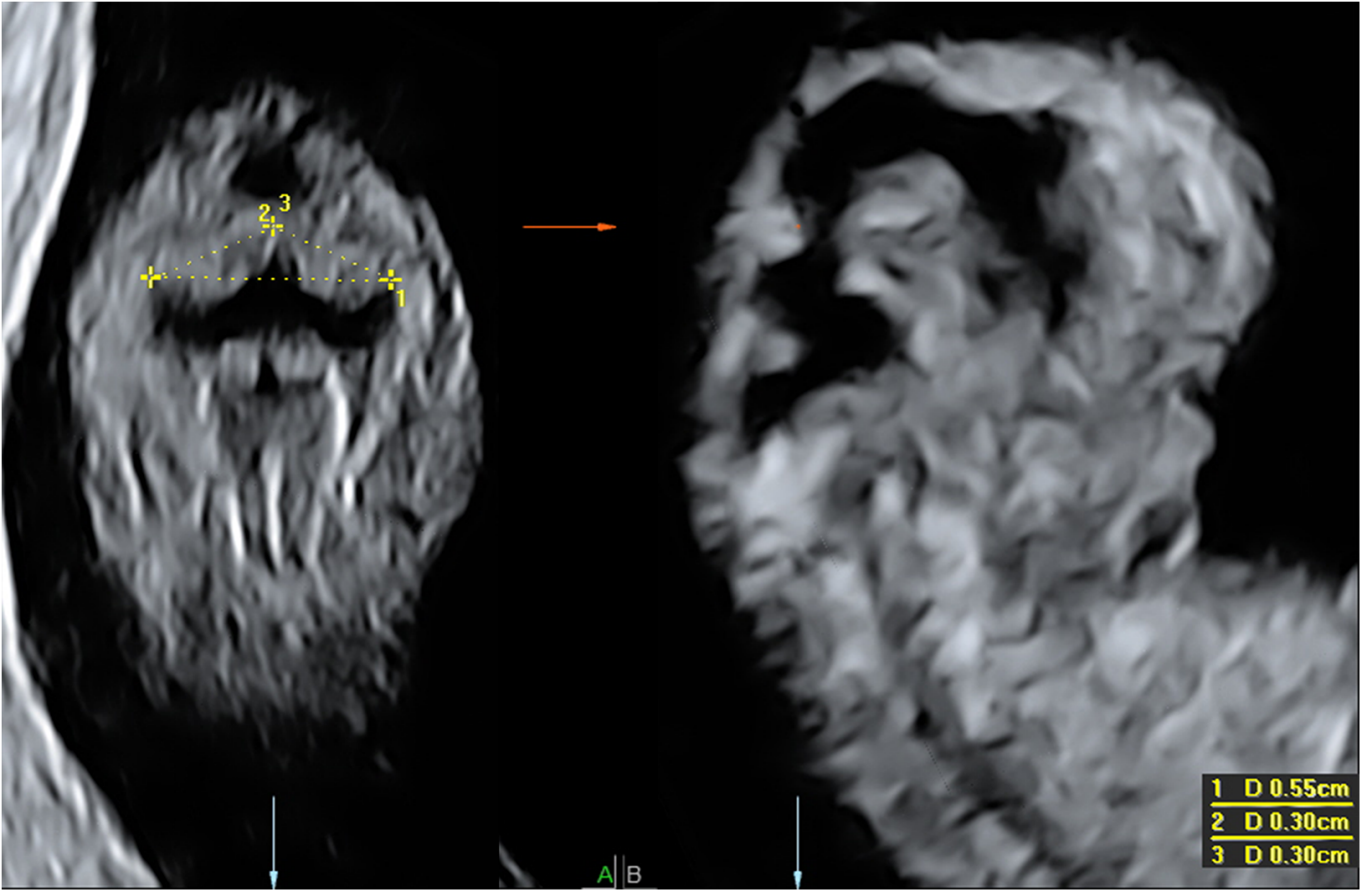
Protocolled display of the three-dimensional ultrasound scan. Three-dimensional ultrasound image of an embryo at 9^+2^ weeks GA with the standardized cerebellum measurements TCD (1), LCD (2), RCD (3) in 4D view.

High resolution transvaginal three-dimensional ultrasounds were performed weekly between 6^+0^ weeks and 12^+6^ weeks of gestation using a 4.5–11.9 MHz transvaginal probe of a Voluson E8 system (GE Medical Systems, Zipf, Austria). Series of three-dimensional ultrasound sweeps were obtained for biometry measurements focusing on the whole embryo or the head separately. Scans were evaluated off-line using specialised 3D-software (4D View, version 7.0, GE Medical Systems). Only high quality images of the fossa posterior in which the cerebellum could be clearly demarcated were accepted for further analysis. We selected only three-dimensional ultrasound scans between 8^+0^ weeks and 12^+6^ weeks gestation, as success rates of cerebellar measurements before 8 weeks GA were very low [6]. Three-dimensional ultrasound scans were displayed in the orthogonal multiplanar mode in a standardized format ensuring that the left and right side corresponded to the viewer’s perspective (Fig 1). Cerebellar measurements were performed in a coronal section of the head at the level of the rhombencephalon enabling the visualisation of cerebellum, fourth ventricle and choroid plexus of the fourth ventricle as previously described [6]. In order to obtain a clearly defined image of the cerebellum at 12 weeks GA, the coronal plane was rotated slightly over the x-axis. The following cerebellar measurements were performed: Transcerebellar diameter (TCD) and left and right cerebellar diameters (LCD, RCD). The ‘distance two points’ function was used to measure the greatest diameter. All measurements were repeated three times in all eligible 3D sweeps, the mean of these measurements was used for analyses. The proportional cerebellar growth was calculated for all cerebellar parameters by dividing the parameter by CRL.

Two observers (IG and AG) measured the cerebellar parameters in the dataset conducted between 2009 and 2010. Inter-observer reliability and agreement were calculated for 35 cerebellar measurements on randomly selected 3D volumes independently repeated by both measurers. Intra-observer reliability and agreement was evaluated by repeating measurements in 30 randomly selected volumes. Reliability calculations included measurements from all gestational ages, with a minimum of 5 scans per gestational week. The observers were blinded to each other’s and previous results.

### Statistical analysis

Statistical analysis was performed using SPSS software version 21.0 (SPSS for Windows, SPSS Inc., Chicago, Illinois, USA) and SAS software version 9.3 (SAS Institute Inc., Cary, NC, USA). Results with p-values of 0.05 or lower were considered statistically significant. General characteristics were calculated for all pregnancies and compared between preconceptional and postconceptional folic acid supplement users. Continuous data of maternal age, BMI, birth weight and GA at birth were compared using the Mann Whitney U-test. Categorical data of ethnicity, education, primigravida, nulliparous, mode of conception, periconceptional use of tobacco and alcohol and infant gender were compared using the Pearson Chi-square test.

To assess the association between timing of initiation of folic acid supplement use during the periconceptional period and first trimester cerebellar growth trajectories we performed linear mixed model analyses. The linear mixed model takes into account the existing correlation between the measurements within one pregnancy. Interaction terms of the timing of initiation of folic acid supplement use with potential confounders were studied, but not included in the model as they yielded no significant contribution. A random intercept only was used to model the within subject correlation, a random slope did not improve the model fit. An unadjusted mixed model was estimated using TCD, LCD and RCD measurements as response variables and CRL and GA as predictors. Timing of initiation of folic acid supplement use was used as independent predictor for cerebellar growth. For the adjusted model we used maternal age, mode of conception, parity, preconceptional smoking, periconceptional alcohol use, BMI, ethnicity, and the measurer as potential confounders, based on literature and significant differences between the preconceptional and postconceptional user groups [13,30]. Stepwise backwards elimination of variables with p>0.20 was conducted to determine the final model.

Additionally, quartiles of red blood cell folate were calculated and used to perform linear mixed models investigating the association with cerebellar growth trajectories. Including the interaction term between red blood cell folate and GA we were able to study the slope between the four quartiles using the third quartile (Q3) as reference [12].

## Results

In 2009 and 2010, 259 viable singleton pregnancies were eligible for this study. We excluded 2 pregnancies conceived from oocyte donations, 43 miscarriages, 1 ectopic pregnancy, 12 pregnancies dated by CRL, 8 pregnancies with abnormal foetal outcome and 7 pregnancies with missing data. This resulted in a final evaluation of 186 pregnancies of 186 women. In 135 (73%) of all pregnancies scans were eligible for cerebellar measurements. All 135 women started folic acid supplements before or after conception. Only 7 women were administered folic acid in a high dosage of 5mg/day, 4 in the preconceptional users and 3 in the post conception users.

In Table 1 the baseline characteristics of the total group of pregnancies, pregnancies with measurements, and the groups of preconceptional and postconceptional initiation of folic acid supplement use are depicted. No significant differences were found between pregnancies with and without cerebellar measurements, except for BMI. Comparing preconceptional and postconceptional folic acid users, general characteristics showed significant differences in ethnicity, primigravida, nulliparous, periconceptional alcohol use, periconceptional smoking and mode of conception via IVF/ISCI procedures. Red blood cell folate is significantly higher in the preconceptional folic acid user group (p<0.01). Education level, BMI, neonatal birth weight, GA at birth and gender were comparable between both groups.

**Table 1:** General Characteristics of the study groups.

Characteristics	All pregnancies(n = 186)	Pregnancies with measurements (n = 135)	Preconceptional Folic acid users (n = 108)	Postconceptional Folic acid users (n = 27)
Maternal				
Age, years	32.1 (18.9–42.7)	32.1 (18.9–42.3)^1^	32.1 (20.3–42.1)	32.7 (18.9–42.3)
Ethnicity				
Dutch	144 (77.8)	99 (73.3)^2^	84 (77.8)	15 (55.5)*
Western-other	16 (8.6)	14 (10.4)	11 (10.2)	3 (11.1)
Non-western	25 (13.5)	20 (14.8)	11 (10.2)	9 (33.3)
Education				
Low	15 (8.5)	12 (8.9)^3^	10 (9.3)	2 (7.4)
Intermediate	54 (30.5)	40 (29.6)	31 (28.7)	9 (33.3)
High	108 (61.0)	77 (57.0)	63 (58.3)	14 (51.9)
BMI, kg/m^2^	23.8 (18.6–38.3)	23.5 (18.6–34.9)**	23.2 (18.6–34.9)	24.1 (19.1–28.7)
Primigravida	69 (37.1)	48 (35.6)	45 (41.7)	3 (11.1)**
Nulliparous	119 (64.0)	83 (61.5)	73 (67.6)	10 (37.0)**
Periconceptional use of alcohol	85 (45.7)	67 (49.6)	49 (45.4)	18 (66.6)*
Periconceptional smoking	31 (16.7)	24 (17.8)	14 (12.9)	10 (37.0)**
Red blood cell folate, nmol/L	1500 (814–2936)	1537 (814–2936)	1626 (844–2936)	1063 (814–1815) **
Mode of Conception IVF/ICSI	57 (30.6)	40 (29.6)	40 (37.0)	0 (0)**
New-born outcome				
Birth weight, grams	3378 (450–4700)	3430 (1715–4700)	3410 (1715–4700)	3440 (2150–4110)
Gestational age at birth, days	276 (187–294)	276 (228–294)	277 (228–294)	276 (258–291)
Gender, male	88 (47.3)	62 (45.9)	50 (46.3)	12 (44.4)

Continuous data is presented as median, range, categorical data as n (%). BMI, body mass index; IVF/ICSI, in vitro fertilization with or without intra-cytoplasmic sperm injection; Missing data was due to incomplete questionnaires. Missing

^1^ n = 3

^2^ n = 2

^3^ n = 6

* p<0.05

** p<0.01

A total number of 880 three-dimensional ultrasounds between 8^+0^ weeks and 12^+6^ weeks gestation were available for detailed cerebellar measurements. The number of measurements per pregnancy is shown in table 2. Cerebellar measurements could be performed in 263 volumes, demonstrating an overall success rate of 29.9%, with the highest success rate of 41.0% in gestational week 8. Table 3 shows the mean of all measurements per gestational week with the corresponding SD values, and the success rate per gestational week.

**Table 2:** 3D-Ultrasound data and the success rate of measurements.

	TCD	RCD	LCD
**Images**	880	880	880
**Measurements**	263 (29.9)	259 (29.4)	259 (29.4)
**Pregnancies with measurements**	135	135	135
**≥ 2 measurements**	77 (57.1)	75 (55.6)	75 (55.6)
**1 measurement**	58 (42.9)	60 (44.4)	60 (44.4)
**2 measurements**	37 (27.4)	36 (26.7)	36 (26.7)
**3 measurements**	31 (22.9)	31 (22.9)	31 (22.9)
**4 measurements**	7 (5.1)	6 (4.4)	6 (4.4)
**5 measurements**	2 (1.5)	2 (1.5)	2 (1.5)

Presented is the number of images (%) eligible for measurement per pregnancy.

TCD, transcerebellar diameter; LCD, left cerebellar diameter; RCD, right cerebellar diameter.

**Table 3:** Measurements per gestational week, with corresponding mean and SD values.

Ultrasound characteristics	Number of measurements	CRL, mm	TCD, mm	RCD, mm	LCD, mm
GA 8 weeks	73/178 (41%)	18.65 (3.44)	4.38 (0.74)	2.58 (0.35)	2.58 (0.35)
GA 9 weeks	70/177 (40%)	26.15 (4.50)	5.56 (0.97)	3.06 (0.43)	3.07 (0.47)
GA 10 weeks	44/178 (25%)	36.45 (5.82)	7.18 (1.17)	3.78 (0.56)	3.79 (0.54)
GA 11 weeks	39/181 (22%)	48.43 (6.70)	8.97 (1.03)	4.62 (0.48)	4.61 (0.57)
GA 12 weeks	37/166 (22%)	61.46 (6.95)	10.34 (1.16)	5.20 (0.57)	5.21 (0.58)

Presented are the number of images eligible for analysis relative to the total number and the cerebellar parameters per GA. CRL, crown-rump length; TCD, transcerebellar diameter; LCD, left cerebellar diameter; RCD, right cerebellar diameter; GA, gestational age.

Intra-observer reliability analysis showed no significant differences in the mean cerebellar parameters (TCD = 0.020mm, 95%CI = -0.054 to 0.094, LCD = 0.007mm, 95%CI = -0.043 to 0.057, RCD = 0.030mm, 95%CI = -0.027 to 0.087). ICC values for all parameters were above 0.99, representing an excellent reliability between measurements. Analysis of the inter-observer agreement showed a significant mean difference between the two measurers (TCD = 0.501mm, 95%CI = 0.260 to 0.742, LCD = 0.310mm 95%CI = 0.134 to 0.485, RCD = 0.200mm 95%CI = 0.063 to 0.337). A good reliability was established with ICC for TCD, LCD and RCD of respectively 0.92, 0.84, 0.89.

Table 4 depicts the results of both crude and adjusted linear mixed model analyses for all 3 cerebellar diameters and the proportional cerebellar growth trajectories by the timing of initiation of folic acid supplement use. Maternal age, mode of conception, periconceptional smoking, periconceptional alcohol use, ethnicity and the measurer were designated confounders in the final model. Subsequently, models for TCD, LCD, RCD and proportional growth (cerebellar diameters divided by CRL) were all adjusted for these final confounders. Both crude and adjusted linear mixed models showed small but significant effects on cerebellar size and proportional cerebellar growth by the timing of initiation of folic acid supplement use as function of CRL Repeating the same analysis for all cerebellar diameters and the timing of folic acid supplement use initiation as a function of GA, no significant differences were observed in either crude or adjusted models. Both crude and adjusted models showed significantly increased proportional cerebellar growth for all three cerebellar diameters when folic acid supplement use was initiated preconceptionally.

**Table 4:** Preconceptional compared to postconceptional initiation of folic acid supplement use and embryonic cerebellar size and growth trajectories as function of gestational age and crown-rump length.

Model		β (se)	95% CI	p-value
**TCD vs. CRL**	Crude	0.286 (0.098)	0.090; 0.482	0.005
	Adjusted	0.257 (0.117)	0.023; 0.491	0.032
**LCD vs. CRL**	Crude	0.153 (0.057)	0.038; 0.268	0.010
	Adjusted	0.171 (0.067)	0.038; 0.305	0.013
**RCD vs. CRL**	Crude	0.132 (0.054)	0.024; 0.240	0.018
	Adjusted	0.156 (0.062)	0.032; 0.280	0.015
**TCD vs. GA**	Crude	0.112 (0.142)	-0.174; 0.398	0.436
	Adjusted	-0.078 (0.154)	-0.388; 0.232	0.616
**LCD vs. GA**	Crude	0.093 (0.069)	-0.045; 0.232	0.183
	Adjusted	0.041 (0.078)	-0.115; 0.197	0.601
**RCD vs. GA**	Crude	0.048 (0.068)	-0.090; 0.185	0.490
	Adjusted	0.008 (0.076)	-0.144; 0.159	0.918
**TCD/CRL ratio vs. GA**	Crude	0.015 (0.004)	0,007; 0,024	0.001
	Adjusted	0.015 (0.005)	0.005; 0.024	0.003
**LCD/CRL ratio vs. GA**	Crude	0.010 (0.003)	0.004; 0.016	0.002
	Adjusted	0.012 (0.003)	0.005; 0.018	0.001
**RCD/CRL ratio vs. GA**	Crude	0.010 (0.003)	0.004; 0.015	0.001
	Adjusted	0.011 (0.003)	0.005; 0.017	0.001

Crude and adjusted linear mixed models comparing effect estimates of preconceptional with postconceptional folic acid use of cerebellar growth trajectories. Estimates are depicted as embryonic cerebellar measurement (TCD, LCD and RCD) in mm per mm increase in CRL or days increase in GA. Crude model: Unadjusted model. Adjusted model, adjusted for designated confounders after stepwise backward elimination. TCD, transcerebellar diameter; LCD, left cerebellar diameter; RCD, right cerebellar diameter; CRL, crown-rump length; GA, gestational age; CI, confidence interval.

**Fig 2:**
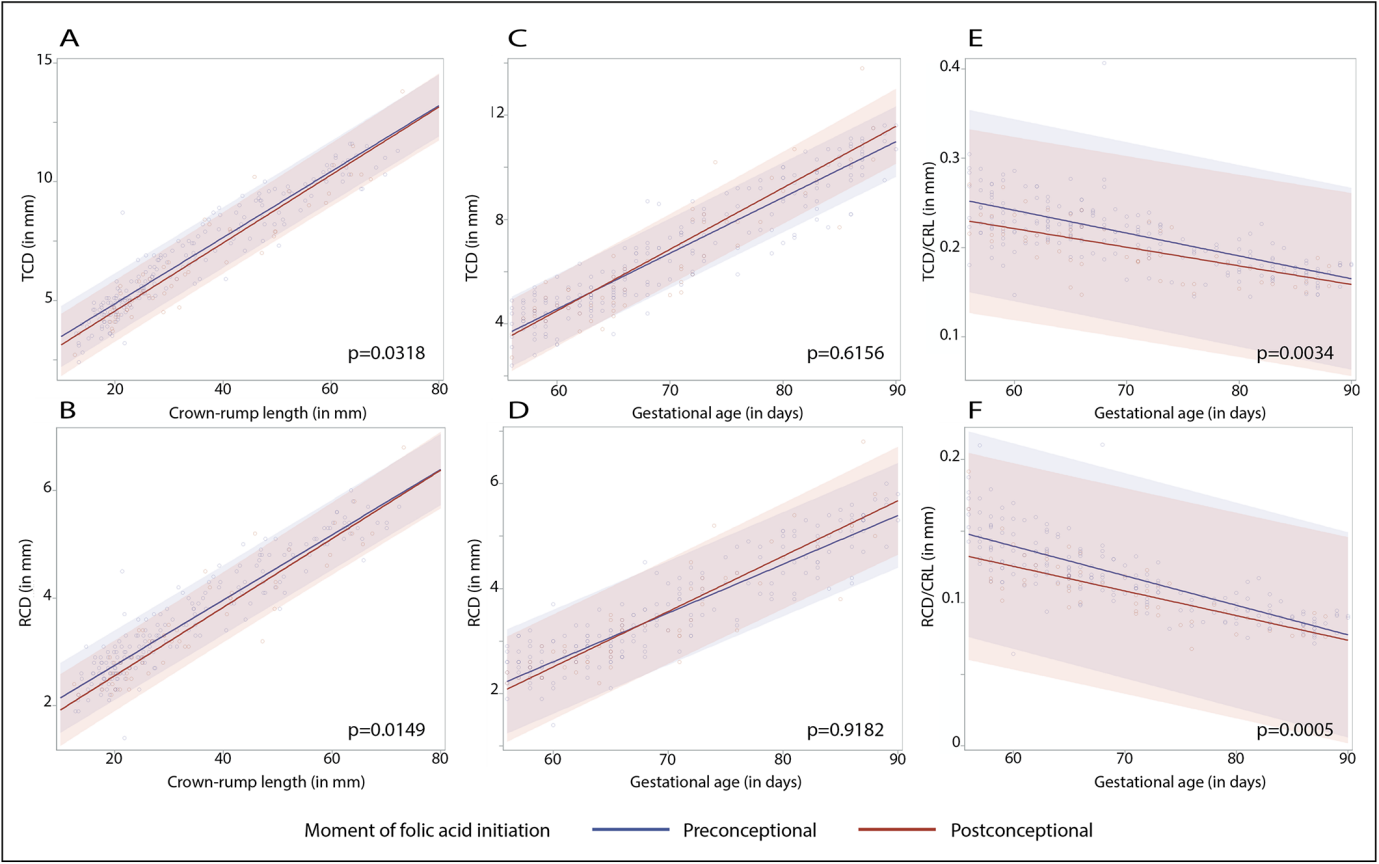
Longitudinal cerebellar measurements and proportional growth by folic acid initiation. Adjusted linear mixed models representing the effects of preconceptional compared to postconceptional initiation of folic acid supplement use on cerebellar size and growth trajectories as function of CRL in millimetres (A and B) and GA in days (C and D) and proportional cerebellar growth trajectories (E and F).

In Fig 2 we graphically display the results of the adjusted linear mixed models as regression lines. The coloured area represents the ninety-five percent prediction interval for both lines. Comparable results for the right and left cerebellar hemisphere were depicted; therefore we only display the TCD and RCD graphs. There is a clear linear display of data points showing slightly increased cerebellar diameters as a function of CRL for TCD (A) and RCD (B). The linear functions of preconceptional and postconceptional initiation of folic acid supplement use as function of GA cross for both the total (C) and unilateral (D) cerebellar diameters. Fig 2E and 2F show the proportional growth trajectories as function of GA, with the associated prediction interval, which decreases with advancing gestation.

**Fig 3:**
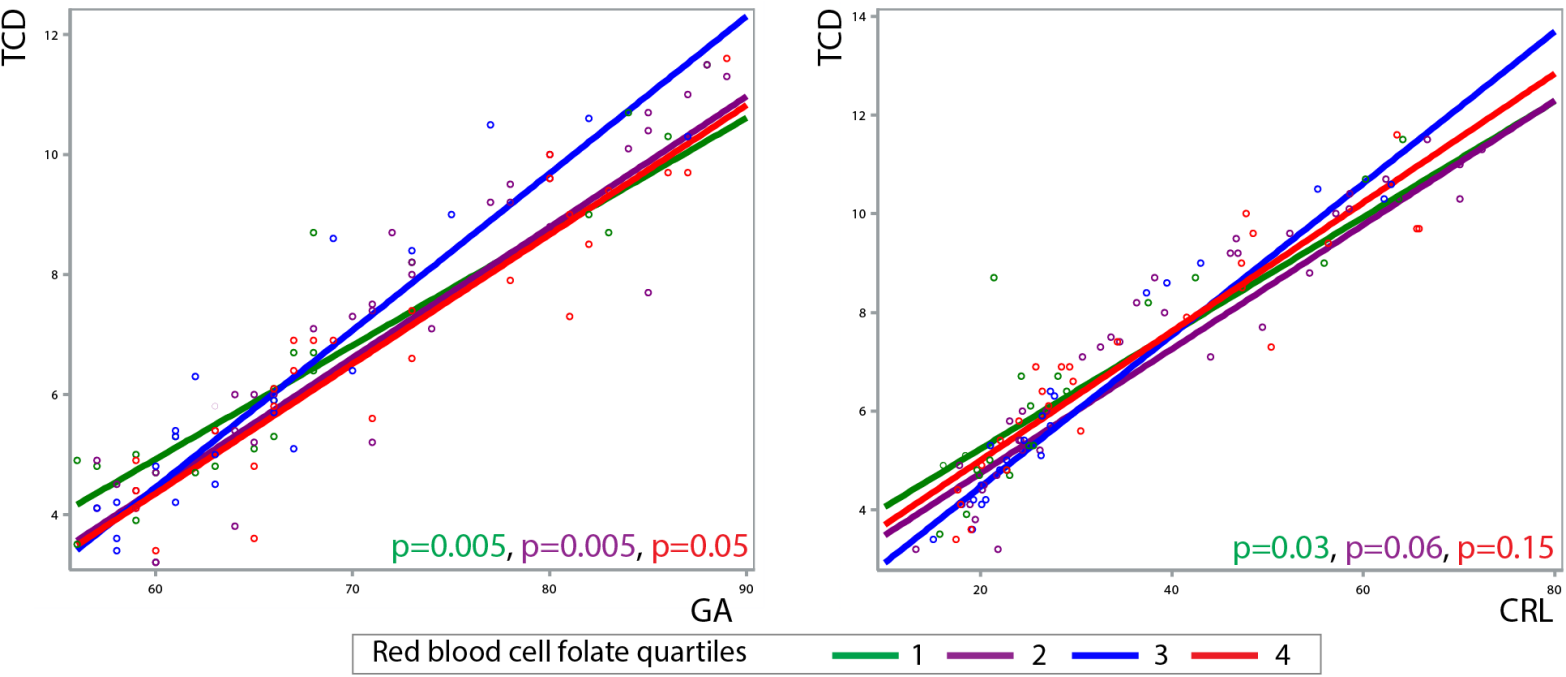
Associations between periconceptional red blood cell folate quartiles and cerebellar growth trajectories. Linear mixed models representing the effects of red blood cell folate quartiles on cerebellar growth trajectories as a function of CRL in millimetres and GA in days. The blue line represents the third quartile used as reference.

In our subgroup of 57 patients quartiles for red blood cell folate were calculated (Q1: 814–1242nmol/L, Q2: 1243–1537nmol/L, Q3: 1538–1813nmol/L and Q4: 1814–2936nmol/L). Linear mixed models showed the highest cerebellar growth throughout all models of total, left and right cerebellar diameter in the third quartile of red blood cell folate levels compared to lowest first and second and highest fourth quartiles as a function of GA (Q1 = -0.0721mm/day, 95%CI = -0.119 to -0.0250, p< 0.01; Q2 = -0.0438 mm/day, 95%CI = -0.0865 to -0.0011, p< 0.01; Q4 = -0.0459 mm/day, 95%CI = -0.0927 to 0.0010, p = 0.05) and CRL (Q1 = -0.0364mm/mm, 95%CI = -0681 to -0.0047, p< 0.05; Q2 = -0.0280mm/mm, 95%CI = -0.0568 to 0.0008, p> 0.05; Q4 = -0.0232mm/mm, 95%CI = -0.0554 to 0.0089, p> 0.05). The same accounts for proportional cerebellar growth which was the highest in Q3 (Q1 = -0.0017mm/mm/day, 95%CI = -0.0039 to 0.0001, p>0.05; Q2 = -0.0010mm/mm/day, 95%CI = -0.0026 to 0.0006, p>0.05; Q4 = -0.0459 mm/day, 95%CI = -0.0018 to -0.0001, p< 0.05). Fig 3 depicts cerebellar growth trajectories associated with the four quartiles of maternal red blood cell folate levels.

## Discussion

This study shows increased, albeit slightly, embryonic cerebellar diameters and proportional cerebellar growth trajectories in ongoing non-malformed pregnancies in a tertiary hospital setting when maternal folic acid supplement use was initiated before conception compared to post conception. Hereby, we discriminate between overall embryonic growth and embryonic cerebellar growth. This data are validated in a subgroup by maternal red blood cell folate levels before 8 weeks GA which is a more reliable marker for periconceptional folate status. It is intriguing that the third quartile of red blood cell folate levels (1538–1813 nmol/L) seems to be the optimum for embryonic cerebellar growth of which the implications for fetal cerebellar growth and future neurodevelopmental outcome needs further investigation. This first study investigating associations between periconceptional folate status and human embryonic cerebellar size and growth trajectories may substantiate to previous animal studies showing alterations in cerebellar growth and development in a folic acid deficient environment [27,31,32]. Besides, our results are in agreement with previous human studies demonstrating significant positive effects of periconceptional maternal folic acid supplement use and folate status on embryonic and fetal size [12,20,30] and DNA methylation of the growth gene IGF2 DMR [33,34].

By establishing a prospective dataset of serial first trimester three-dimensional ultrasound images together with periconceptional exposure data and follow-up information, we were able to study associations between periconceptional folic acid supplement use and embryonic cerebellar growth. We substantiate the validity of our questionnaire data by verification at the intake visit and measurements of red blood cell folate concentrations in a subgroup. Although data on the precise day of folic acid initiation was not available, red blood cell folate levels were significantly higher when the timing of initiation of folic acid supplement use was defined as preconceptional. This agrees with the assumption that the timing of folic acid use initiation serves as measure for periconceptional folate status which may involve the embryonic environment as well. First trimester red blood cell folate levels follow an optimum curve in which the third quartile is associated with the highest cerebellar growth rates compared to the first two and upper quartile which corresponds to the available evidence of its effects on overall embryonic growth [12]. Because of the prospective and observational character of our study we were able to study associations, causality however could not be shown, since this warrants a randomised controlled trial.

The low success rates of the cerebellar measurements are comparable to previous publications and are due to non-targeted scanning for the purpose of embryonic biometry and volumetric measurements, motion artefacts, acoustic shadowing and maternal factors, such as BMI and an unfavourable position of the uterus [6]. Although differential confounding may be an issue, it seems unlikely as these factors are unrelated to the selected population, the timing of folic acid initiation, red blood cell folate levels and cerebellar outcome. Low success rates could compromise the longitudinal setting of the study. Yet, 57% of the patients had 2 or more measurements. Since measurements within subjects are strongly correlated, linear mixed models are the most appropriate method for the statistical analyses of our data. Preconceptional and postconceptional folic acid users are in many ways not totally comparable. Hence, for statistical analyses we adjusted for known confounders of embryonic growth and maternal characteristics to minimize confounding. Demonstrating comparable effects in both crude and adjusted models for all cerebellar diameters strengthened the model fit.

Differences in cerebellar size and proportional growth trajectories may be explained biologically by folates’ vital role in synthesis of DNA, RNA and proteins for cellular growth and differentiation processes. It could be hypothesized that in early embryonic development epigenetic modification of genes including those regulating the development of the cerebellum as part of the central nervous system is altered by the use of folic acid amongst other factors [34,35]. Also, the proportion of cerebellar size in relation to CRL increased when folic acid use was initiated before conception. This is either the result of a larger cerebellum, a smaller CRL, or a combination of the two. Since previous literature demonstrated larger embryonic and fetal biometric measurements in association with optimal folate status, proportional cerebellar growth seems to be increased due to a relatively larger cerebellum in particular [12,30]. However we did not find significant differences in cerebellar measurements between preconceptional and postconceptional folic acid users using GA as predictor. We assume that this is the result of inaccurate pregnancy dating, despite using strict criteria based on reliable LMP only. Inaccurate determination of GA is a recurrent issue in embryonic and foetal growth studies [30]. In this context, it is important to consider that our methodology for pregnancy dating is stricter than methods used routinely for clinical practice. Hence, our method enables designation of very early growth deviations corrected by pregnancy dating using CRL.

Prenatal modification of cerebellar growth potentially has consequences for neurodevelopmental outcome [14,15]. One could speculate that early changes in embryonic size affect neurodevelopmental functions in early and later life. However, exploring the association between the small differences of embryonic cerebellar growth and its possible clinical implications is beyond the scope of this study. This warrants larger periconceptional cohorts including biomarkers and pre- and postnatal neuroimaging combined with long term standardized neurodevelopmental follow-up. Also, considering the tertiary hospital setting, external validity of our results should be studied in a general population cohort.

In conclusion, our findings show small albeit significant associations between preconceptional initiation compared to postconceptional initiation of folic acid supplement use, used as measure of folate status in the embryonic environment, and increased size and growth trajectories of the embryonic cerebellum. Although the effects of these small differences for prenatal and postnatal neurodevelopment are unknown, this study further supports the importance of periconceptional maternal folic acid supplement use.

## Supporting Information

S1 File Minimal data set. (SAV)
