# Correction: Periconception Maternal Folate Status and Human Embryonic Cerebellum Growth Trajectories: The Rotterdam Predict Study

**DOI:** 10.1371/journal.pone.0203983

**Published:** 2018-09-11

**Authors:** Irene V. Koning, Irene A. L. Groenenberg, Anniek W. Gotink, Sten P. Willemsen, Manon Gijtenbeek, Jeroen Dudink, Attie T. J. I. Go, Irwin K. M. Reiss, Eric A. P. Steegers, Régine P. M. Steegers-Theunissen

## Notice of republication

An incorrect version of S1 File was published in error. This article was republished on August 29, 2018 to correct for this error. Please download this article again to view the correct version.

## References

[1] Koning IV, Groenenberg IAL, Gotink AW, Willemsen SP, Gijtenbeek M, Dudink J, et al (2015) Periconception Maternal Folate Status and Human Embryonic Cerebellum Growth Trajectories: The Rotterdam Predict Study. PLoS ONE 10(10): e0141089 10.1371/journal.pone.0141089 26491876 PMC4619586

